# Lessons Learned from the Studies of Roots Shaded from Direct Root Illumination

**DOI:** 10.3390/ijms222312784

**Published:** 2021-11-26

**Authors:** Jozef Lacek, Judith García-González, Wolfram Weckwerth, Katarzyna Retzer

**Affiliations:** 1 Laboratory of Hormonal Regulations in Plants, Institute of Experimental Botany, Czech Academy of Sciences, 165 02 Prague, Czech Republic; lacek@ueb.cas.cz (J.L.); garciago.judith@gmail.com (J.G.-G.); 2 Department of Experimental Plant Biology, Faculty of Science, Charles University, 128 00 Prague, Czech Republic; 3 Department of Functional and Evolutionary Ecology, Molecular Systems Biology (MoSys), Faculty of Life Sciences, University of Vienna, Djerassiplatz 1, 1030 Wien, Austria; wolfram.weckwerth@univie.ac.at; 4 Vienna Metabolomics Center (VIME), University of Vienna, Djerassiplatz 1, 1030 Wien, Austria

**Keywords:** D-rootsystem, direct root illumination, root growth, reactive oxygen species, flavonols, abiotic stress, light escape mechanism, auxin, cytokinin, dark-grown roots

## Abstract

The root is the below-ground organ of a plant, and it has evolved multiple signaling pathways that allow adaptation of architecture, growth rate, and direction to an ever-changing environment. Roots grow along the gravitropic vector towards beneficial areas in the soil to provide the plant with proper nutrients to ensure its survival and productivity. In addition, roots have developed escape mechanisms to avoid adverse environments, which include direct illumination. Standard laboratory growth conditions for basic research of plant development and stress adaptation include growing seedlings in Petri dishes on medium with roots exposed to light. Several studies have shown that direct illumination of roots alters their morphology, cellular and biochemical responses, which results in reduced nutrient uptake and adaptability upon additive stress stimuli. In this review, we summarize recent methods that allow the study of shaded roots under controlled laboratory conditions and discuss the observed changes in the results depending on the root illumination status.

## 1. Introduction

Plants have evolved a finely tuned network of signaling pathways to simultaneously adapt to multiple, continuously occurring changes in environmental conditions [1,2,3,4]. Environmental changes can either affect shoot or root development locally or influence the development and growth behavior of the entire plant [1,2]. External stimuli range from changing light conditions that affect the photosynthetic activity and serve as a signal to control organ growth, to energy-consuming responses to abiotic and biotic stresses that challenge plant productivity [1,5,6]. Plants are very flexible and have an amazing ability to change cell shape and tissue organization to respond to exogenous stimuli [7,8,9,10,11]. To ensure efficient plant growth and productivity we need to understand how plants adapt under less beneficial environmental conditions. Plants are divided into the aboveground located shoot and the underground located root [2,4]. The shoot produces energy in the form of carbohydrates via photosynthesis, wherefore its productivity primarily depends on light quality and intensity [4,6,12,13,14]. Plant productivity also depends on the delivery of water and nutrients from the soil, which are taken up over the root [4,15]. The root needs to navigate through the soil towards beneficial areas rich in water and nutrients, but to avoid obstacles, also in the form of toxic compounds [16,17,18]. To orchestrate root development and the modulation of directional root growth, plants have evolved cellular mechanisms that rely on the spatially and temporally regulated distribution of signaling molecules, including sugars, phytohormones, and Reactive Oxygen Species (ROS) [4,9,18,19,20,21,22,23,24]. Direct root illumination not only affects root growth alone but leads to altered communication between shoot and root, which also modulates the distribution of signaling molecules and eventually reduces efficient nutrient uptake, and negatively affects the ability of plants to adapt to additive stress stimuli [6,9,25,26]. In this review, we summarize recent findings showing how established laboratory growth conditions, which include plant cultivation on growth medium with roots exposed to light, affect plant development and root responses to abiotic stresses.

## 2. Standard Laboratory Conditions and Their Effects on Root Growth

**Figure 1:**
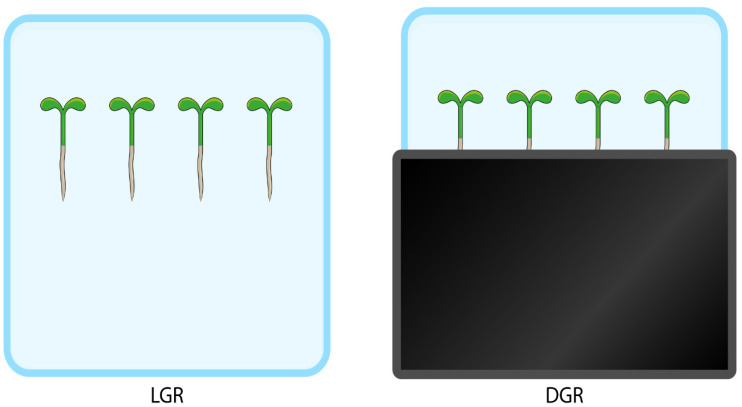
Schematic representation of the principle of light versus dark-grown roots. To shade roots from direct illumination, a cover is used, often made of methacrylate or black cardboard. A video showing how to assemble the so-called D-root system, which is widely used in the D-root community studying *Arabidopsis thaliana* and was designed by the del Pozo laboratory, is available online https://www.researchgate.net/publication/281436423_Assembling_the_D-root_system (accessed on 23 November 2021) [26].

Environmental conditions, on the one hand, shape the overall architecture of the plant, but also determine its ability to cope with additive stress stimuli at the level of individual organs down to the cells [3,9,23,26,27,28,29,30]. Roots have evolved to anchor the plant in the soil and also to absorb water and minerals from it [3,4] Furthermore, roots developed to direct the growth direction along the gravity vector and to avoid direct illumination [9,26,30]. As early as 1880, Darwin and Darwin suggested in their study ´The Power of Movement in Plants´ directional root growth depending on growth conditions [31]. Like all land species, terrestrial plants developed mechanisms to orient themselves along the gravity vector [18]. However, negative phototropism of the root, the active root growth away from light, is also mediated in the very root tip [32]. Therefore, the conditions under which the plants are grown must be taken into account, as they can lead to additive responses or even mask phenotypes [9,26,29,30,33]. To make roots accessible for microscopy, phenotypic analysis, cell biological, and biochemical approaches, seedlings are grown in laboratories on plates with roots exposed to light (Figure 1), often in combination with sugar supplementation to enhance seedling growth [24,26,29,30]. While these standardized laboratory growth conditions may seem convenient and practical at first glance, they have recently been shown to have profound effects on root growth, architecture, root hair emergence and elongation, nutrient uptake, and ultimately whole plant growth [26,28,29,30]. In particular, the combination of light exposure of roots and exogenous sugar supplementation, usually sucrose or glucose, resulted in the biggest changes in root growth and responses to other exogenous stimuli compared to non-illuminated roots [24,29,30]. Sugars, as products of photosynthesis in the leaves, are actively distributed to the roots, where they not only function as building blocks for new molecule biosynthesis and energy source to enhance cellular activity, but also as signaling molecules [4]. By combining exogenous carbon sources and phytohormones with different illumination conditions for shoot and root, it became clearer to what extent the signaling cascades are interdependent and even overlap each other [26,29,30,34].

The regulation of root growth (e.g., the timing of lateral root and root hair emergence, directional root growth) is highly dependent on the finely tuned distribution of numerous signaling molecules, including sugars, ROS, phytohormones, and other small molecules whose availability per cell is strongly modulated by internal and external conditions [4,16,19,29,33,35,36,37]. Therefore, the direct interplay of phytohormones, light, and sugar signaling pathways in roots exposed to direct illumination compared to shaded roots has been the target of several recent studies [4,21,23,24,26,27,28,32,37,38,39,40]. Overlapping results show particularly striking differences in ROS production and distribution along the root, but also changes in phytohormone regulated responses have been reported [9,27,38,41]. 

The number of studies addressing the effects of direct root illumination on the outcome of stress response experiments, nutrient uptake, shoot–root communication, and the ability to modulate root architecture and directional root growth, is steadily increasing. In addition to efforts to track root growth directly in soil [42], several experimental setups have been described, which allow cultivation of seedlings in plates on sterile medium, but shade the roots from direct light exposure [43]. The reasons behind the efforts to keep roots growing under more natural light conditions, but still on a sterile medium, result from the wish to prepare plant material for microscopy studies, phenotypic analysis, and further molecular biological studies. These approaches range from optimized medium preparation with charcoal [41] to the use of the so-called D-root system, which has recently become very popular [26]. Laboratories either use the D-root box available for purchase, as first described by Silva-Navas et al., 2015 [26,27,28,29,30,33], adaptations to study root illumination responses [6,44] or versions made of black cardboard [36,45]. However, regardless of the method used to shade the roots from direct root illumination, the results obtained are reproducible. Among the most obvious differences in root trait establishment depending on the illumination status include reduced root growth by direct root illumination due to lower meristematic activity, but increased root elongation rate, also known as root escape mechanism. In addition, seedlings with roots exposed to direct root illumination show an overall lower nutrient uptake capacity and a more sensitive response to abiotic stresses [9,26,27,28,29,30]. Finally, differential root development under direct illumination results in altered shoot growth, as shoots with shaded roots accumulate less mass and anthocyanins, demonstrating differential distribution of resources throughout the plant and fitness depending on the experimental growth conditions chosen [26]. 

## 3. Differences in Root Growth Adaptation Depending on Root Illumination Status

### 3.1. Root Traits Altered by Direct Illumination

Roots are positive gravitropic and negative phototropic, and they evolved to grow into soil shaded from light [4,7,44]. Roots also express photoreceptors, which allows them to adapt directional root growth to avoid direct illumination [3,21]. Furthermore, light perception also results in altered root growth rate, sensitivity to additive stress stimuli, and changes in root system architecture establishment depending on the activated photoreceptor and the illuminated area of the root [1,32,40]. Therefore, roots respond differently to direct root illumination depending on the wavelength or direction of illumination. Comprehensive reviews of the photoreceptors expressed in roots and involved in the modulation of root growth can be found in Zdarska et al., 2015, Silva-Navas et al., 2015 and van Gelderen et al., 2018, among others [3,21,26]. Root meristem activity is modulated upon exogenous and endogenous stimuli [2,4,46,47,48,49,50]. Cell proliferation comes to a halt when environmental conditions are not favorable for the plant, such as when red, energy-intensive light is perceived [9,23,26,32]. After leaving the meristem, cells pass through the transition and elongation zone towards the differentiation zone, where cell fate depends on maturation and growth as well as environmental stimuli. It has been reported that blue light receptors appear to modulate the elongation rate above the meristem [9,26,51]. 

**Figure 2:**
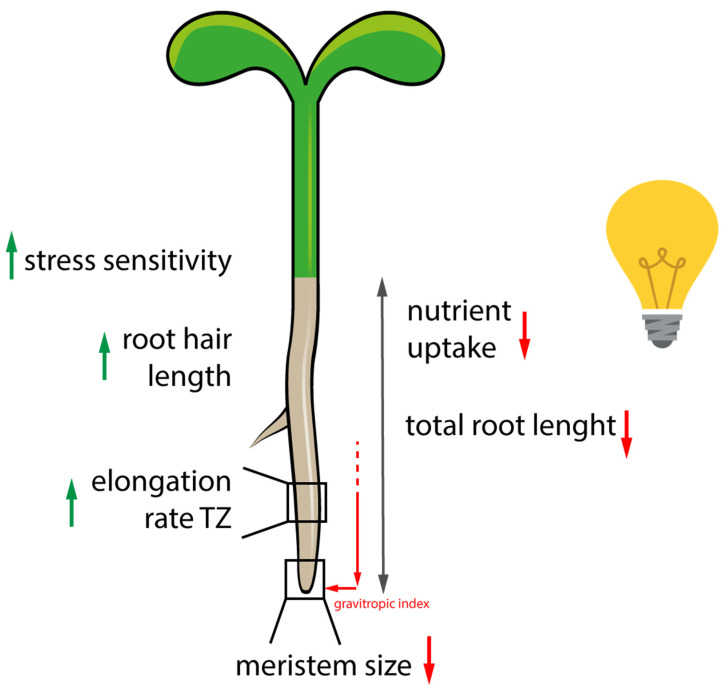
Summary of repeatedly observed root traits, which differ depending on the root illumination status. When roots are shaded from direct root illumination, independent studies from different laboratories confirmed a higher activity of the meristem, better efficiency in nutrient uptake, and better coordinated directional root growth. Furthermore, cell elongation rate in the transition zone (TZ) is reduced, as the light-induced Root Escape Mechanism is missing. Moreover, light-grown roots show enhanced sensibility towards additive stress treatment and cope less efficiently or slower upon stress application, probably due to elevated reactive oxygen species production (ROS), which activates stress response signaling pathways. Elevated ROS levels are suspected further to induce root hair outgrowth and elongation closer to the meristem in light-grown roots.

The application of the D-root system confirmed that the quality of light has an impact on root growth, by studying the effect of individual wavelengths on plants lacking single photoreceptors [26]. Silva-Navas et al., 2015, in their comprehensive study of differences in plant growth between seedlings with light- and dark-grown roots (LGR, DGR), showed that LGR are up to 25% shorter compared to DGR [26]. García-González et al., 2021a, and b, showed that the difference in total root length is more pronounced when sucrose is added to the growth medium [29,30]. When comparing publications that evaluated total root length as a function of root illumination status, it became clear that the difference in root length was less pronounced in seedlings younger than seven days after germination, but was striking from twelve days after germination [26,30] (Figure 2). Direct root illumination also causes root growth to deviate from vertical (Figure 2), which in turn is enhanced by the addition of sugar to the growth medium [24,29]. In addition, the length of root hairs closer to the meristem is increased, which is likely due to the increased concentrations of ROS in LGR [28,29]. Recently, ROS were shown to be critical modulators of root hair elongation and lateral root outgrowth and root growth in general [26,27,40,52,53,54,55,56]. It has also been shown that ROS modulate negative phototropism even upon brief irradiation with blue light of 82 μmol m^-2^ s^-1^ for 10 s, and roots immediately responded by producing ROS in the root tip, which was accompanied by a rapid increase in root growth rate, a phenomenon they termed Root Escape Tropism [9,23,32,40,41].

### 3.2. The Modulation of Root Growth at the Cellular Level Depends Strongly on the Interplay between Reactive Oxygen Species and Flavonols

The root absorbs water and nutrients from the soil, which determines the productivity of the plant [2,4]. Direct illumination of the root results in decreased accumulation of nutrients, including potassium, sodium, and molybdate [26]. Only iron was accumulated in the root and shoot of plants whose roots were exposed to light, and since iron dissolution is modulated by redox reactions [57], photocatalysis of various ROS in LGR could stimulate iron accumulation [26]. In addition, accumulation of ROS also plays an important role in modulating additive stress responses of roots, such as nitrogen deficiency [26]. Total root length and ion accumulation are reduced in LGRs under N deficiency, while DGRs show limited reduction in total root length under N deficiency [26,58,59]. LGRs have an overall increased content of individual ROS [55], but the content of ROS scavengers is also increased, which eventually leads to a decrease of specific ROS compared to DGR, which needs further investigation [26]. ROS scavengers in the form of secondary metabolites, especially flavonols, accumulate in LGR, which are crucial for the proper regulation of root light avoidance [27]. Silva-Navas et al, 2016 described how phototropic reactions in LGR are orchestrated by hydrogen peroxide and cytokinin, which stimulate the accumulation of flavonols along the transition zone to promote cell elongation, even in an asymmetric manner under unilateral root illumination [27]. Under unilateral illumination, roots accumulate flavonols closer to the light source, resulting in asymmetric cell elongation that forces the root to bend away from the light [27]. Blue light is known to activate the PHOTOTROPIN1 (PHOT1) receptor in the root transition zone, where it is associated with modulation of the subcellular distribution of the auxin efflux carrier PIN-FORMED 2 (PIN2) [51,60].

The length of the root is determined by the proliferation rate of the meristem, which is reduced when the root is directly illuminated, and by the elongation rate of the cells, which is stimulated to enable the light escape mechanism in the root [9,27]. It is worth noting that the proliferation rate is suppressed in LGR due to phototoxicity, which has been associated with increased flavonol levels [27]. Meristematic activity is also reduced when the whole plant is shifted into darkness, but the gradual shutdown of root growth results from the reduction of photosynthetic activity of the shoot and changes in shoot–root communication [3] Therefore, data obtained from whole plants shifted to darkness or even etiolated plants should be taken with caution and cannot be compared with results from D-root experiments [30]. Previous studies have already shown that the suppression of meristematic activity in illuminated roots is primarily due to UV-B triggered ROS accumulation [9,55]. Overall, meristematic activity is balanced by ROS accumulation depending on environmental conditions and the ability to produce ROS scavengers to maintain efficient root growth [27,40,49,56,61,62]. The rate of cell elongation in the transition zone of DGR is slower than in LGR or DGR treated with flavonols, while the final cell size in differentiated cells is similar in DGR and LGR [27]. This is also consistent with a study showing that illuminated roots respond more slowly when exposed to a NaCl gradient [9]. Cell expansion depends on the finely tuned establishment of the cytoskeleton, and several studies have shown that direct illumination of roots alters its appearance, including increased actin polymerization and bundling, which could be the reason for altered dynamics of root growth responses of LGR [9,43]. Modulation of the cytoskeleton is responsible for various morphological changes at the cellular and subcellular levels, including the properties of lytic vacuoles, intracellular trafficking, and plasma membrane (PM) responses [33,37,39,63,64,65]. Several changes have been linked to the regulation of auxin distribution in the root, so it is not surprising that responses to hormones are also affected by direct root illumination [21,23,33].

### 3.3. Phytohormones

Phytohormone biosynthesis, distribution, and metabolism, either to store or to degrade them, highly depends on the developmental stage of the plant, but also the availability of resources and growth conditions [2,22]. Shoot and root influence each other in the production of individual hormones and exchange them to promote or inhibit the growth of individual organs or tissues [2]. Since recent studies have shown that the illumination status of individual plant organs has a profound effect on overall plant growth, it is not surprising that LGR and DGR responded differently to exogenous hormone application [26]. Exogenous hormone treatment showed greater inhibition of total root length of DGR compared to LGR when treated with epi-brassinolide (eBl), abscisic acid, the cytokinin 6-benzylaminopurine, the synthetic auxin 2,4-dichlorophenoxy-acetic acid (2,4-D), and the auxin transport inhibitor 1-N-naphthylphthalamic acid [26]. 

The close interplay of auxin transport, biosynthesis, conjugation, perception, and signaling [66,67,68] enables constant plant growth through a balance between cell division and elongation. While total root length was more inhibited by 2,4-D in LGR compared to DGR, the natural auxin indole-3-acetic acid had the opposite effect, suggesting that light attenuates the inhibition of root growth by 2,4-D [26]. Mutants of genes involved in auxin signaling, *auxin resistance protein 1-12* and *transporter inhibitor response 1-1*, developed a higher number of lateral roots in DGR compared with wild type, but total root length was not affected [26]. Root illumination might affect auxin homeostasis, which would explain the differential response to different auxins as well as root growth and morphogenesis, but detailed studies have not yet been completed. So far, preliminary insights have been gained into the modulation of auxin carrier distribution and subcellular localization in response to root illumination status [33,38,51]. Fine-tuned polar auxin transport ensures over long distances an active and regulated transport and gradient establishment on tissue and cellular level, followed by subcellular rearrangements to modulate plant growth [65,68,69]. Auxin efflux carriers have been extensively studied in terms of their intracellular distribution, transport, and posttranslational modifications that modulate their activity and abundance at the PM [16,33,35,70,71], but all of these studies were either done in LGR or in plants that were grown etiolated or dark-shifted. Only recently, a few studies have been published focusing on the distribution of PIN1 and PIN2 in DGR. Direct root illumination results in lower levels of PIN1 in the stele of the root, which have been shown to be modulated post-translationally, as the transcript levels did not change depending on the illumination status of the root [27]. Furthermore, internalization of PIN2:mcherry occurs from the PM towards the lytic vacuole, which is not visible when PIN2 is tagged to a pH sensitive fluorescent protein [27,33,38,39,51]. The intracellular targeting of PIN2 to the lytic vacuole is mediated by ubiquitination, and fusion between PIN2 and a single ubiquitin unit increases the turnover of PIN2 from the PM directly into the lytic vacuole, which can be inhibited by exogenous eBL treatment [33,72]. While PIN2:ubq turnover in LGR can be inhibited by eBL, leading to increased PIN2:ubq levels at the PM, wild-type PIN2 abundance at the PM increases significantly after eBL treatment only in DGR, but no longer in LGR, which is consistent with previous studies suggesting that root illumination leads to stabilization of PIN2 at the PM [33]. In addition, a recent study showed that direct root illumination, which leads to increased root hair outgrowth closer to the meristem in wild-type roots, is reduced in the PIN2 knockout mutant *eir1-4*, consistent with the importance of shoot-directed auxin transport for PIN2-mediated root hair outgrowth [4,29,73]. Regulated shootward auxin distribution has been well described to ensure proper root growth and thus plant nutrition [19,74,75]. This includes processes such as cell division, regulation of cell expansion, directional root growth, root hair outgrowth, and development of lateral roots, which are modulated by finely tuned auxin gradients, thus most studies have targeted auxin-related mechanisms [1,4,76,77]. The regulatory role of auxin distribution and signaling in the root were intensively studied, but the understanding of how exactly auxin distribution and homeostasis are modulated upon distinct environmental conditions is still incomplete [29,37,73,78,79]. Therefore, reducing external stimuli by using the D-root system allows us to dissect better which signaling pathways are in which way interconnected [15,48,80]. It has been shown that direct illumination of roots increases the amount of flavonols, and exogenous application of flavonols results in lower PIN1 levels in the root, which could limit the delivery of auxin from the shoot to the root tip [27]. Flavonols are known to control auxin distribution in the root at multiple levels, including inhibition of auxin transport and modulation of indole-3-acetic acid (IAA) catabolism [81,82,83,84]. The reduction of LGR meristem size could on one hand directly result from lower auxin levels, as auxin drives meristematic activity to a certain extent [85,86], but data showing auxin distribution along the root depending on the illumination status are still outstanding. Alternatively, an altered cytokinin:auxin ratio might modulate the cell fate switch between meristem and transition zone [27,87,88]. Cytokinin itself seems in LGR to induce flavonol biosynthesis via SHORT HYPOCOTYL 2 (SHY2) to reduce auxin transporter abundance and thereby, delivery of auxin to the root tip [27]. This corresponds to previously obtained data that describe how cytokinin and auxin signaling pathways interact to modulate root meristem [27,87,89]. Moreover, the application of the D-rootsystem allowed defining a new role of the cytokinin cis-zeatin in orchestrating root growth adaptation, including regulation of root hair outgrowth, upon phosphate deficiency [28].

Possible, cross-talks in-between individual phytohormonal signaling pathways and responses to root illumination status are outstanding, and could probably reveal regulatory mechanisms that were masked so far. Light perception and root growth modulation are connected over signaling pathways of other hormones too [32], but how far they differ in DGR must be still investigated. For example, BR signaling is induced in roots upon blue light illumination and is known to modulate ROS and ethylene synthesis to restrict root growth [90,91]. Plant roots are very plastic and can adjust their tissue organization and cell appearance during abiotic stress responses, whereby direct root illumination results in changes the sensitivity of plants towards individual external stimuli [26,92].

### 3.4. Additive Stress Responses under Direct Root Illumination

Environmental conditions are constantly changing and plants, as sessile organisms, must often adapt rapidly to stimuli that occur simultaneously [1]. The composition of the soil is also not uniform and can change during root growth. Not only are nutrients unevenly distributed or dissolved, but there may be a sudden accumulation of toxic compounds or a decrease in the availability of water [1,28,49,93]. Environmental pollution and extreme weather conditions affect plant growth by activating multiple response pathways simultaneously, usually leading to reversible growth arrest or even plant death [94]. Direct root illumination triggers additional stress responses that do not occur in DGR; therefore, to understand how roots respond in nature the D-root system or similar advice will provide more accurate results [26]. The establishment of ROS gradients in the root tip is known to be crucial to rapidly modulate root architecture and growth upon stress exposure [26,27,53,55,56]. As elevated ROS levels impair N uptake in roots, which massively affects resource accumulation required for mass production of plants [58], it is not surprising that LGR are shorter than DGR under N deprivation [26]. Phosphate deficiency (Pi) combined with direct root illumination significantly inhibited root meristem activity by up to 50% and stimulated root hair outgrowth closer to the root meristem, whereas root hair elongation was impaired [28]. Total root length was even more impaired of LGR under osmotic or salt stress [26]. Application of osmotic stress in the form of 250mM mannitol inhibited root growth of LGR by 55% but only 44% of DGR and treatment of LGR with 100mM NaCl resulted in 40% shorter roots while DGR where inhibited only by 27% [26]. In addition to a more drastic reduction in root growth of LGR when grown directly on NaCl, LGR also show slower salt avoidance, halotropism, when grown on split plates where NaCl is added only to the lower part of the plate and seedlings are placed on medium without salt [9]. Furthermore, it has been shown that particular irradiation of the root tip with UV-B lowers root response to salinity [9]. Moreover, the UV-B receptor UV RESISTANCE LOCUS 8 (UVR8) is involved in growth reduction under drought, which again shows that multiple stress responses are regulated by overlapping signaling pathways, and additional stress in form of direct root illumination may mask or alter research outcomes [9]. AtUVR8 further complements an osmosensitive *Saccharomyces cerevisiae* mutant and its overexpression in *Arabidopsis thaliana* leads to reduced primary root growth, especially under osmotic and salt stress, which is again associated with flavonoid accumulation [9,95]. On the one hand, lateral root growth seems to be rather promoted under salt stress growth conditions to increase root surface area and search for water and nutrients [1], but it is impaired when the root is exposed to excessive illumination, especially UV-B [9]. Studying the adaptation of root system architecture under toxic growth conditions, including salt stress and drought, is crucial as the availability of unpolluted soil and water for crop production continuously decreases, leading to devastating crop losses [93,96,97,98]. To study plant stress responses in soil, Rhizotrons, also known as Rhizoboxes, are used, which still allow monitoring of root growth under controlled conditions [42,96,99,100]. Results obtained from DGR are more comparable to studies done on soil compared to LGR. Recent studies on the adaptation of pearl millet root growth to drought in so-called high-temperature tubes with sensors at the top and bottom of the tube to monitor water content made it possible to observe changes in the proteome and metabolome during drought stress [96]. Combined evaluation of changes at the proteomic level and metabolomic analysis of root exudates confirmed modulation of auxin homeostasis and linked increased flavonoid production to the observed reduction in root growth [101]. This demonstrates the importance of performing basic research under more natural conditions to allow a smoother transition of findings into applied science. Furthermore, Rhizotrons are commonly used to examine the interaction of roots with the rhizosphere, which is very sensitive to light, and most studies have been conducted on roots grown in soil [100,102]. Therefore, recent attempts to study biotic interactions with the root, which can be beneficial or threatening, include the use of transparent artificial soil for DGR, which in turn will allow a more detailed study of root growth adaptation at the cellular level under more natural conditions [103,104].

## 4. Conclusions

Roots have evolved as below-ground organs of the plant, and direct light illumination triggers stress responses that result in escape growth away from the light source and morphological and cellular changes, including responses to phytohormones and other small molecules that act as signaling molecules, such as ROS and flavonols. Two growth conditions of *Arabidopsis thaliana* seedlings are manifested in the majority of laboratories studying plant development and growth adaptation. One is to grow seedlings on agar plates with roots continuously exposed to light to the same extent as the shoot. Direct root illumination results in altered root architecture, which origins in changes at the subcellular level, including altered auxin carrier abundance and subcellular distribution. The other habit is to add sugar to the growth medium to enhance the growth rate of the seedlings, which has a massive impact on the genetics, molecular biology, metabolomics, etc., of the roots. The triggered responses negatively affect nutrient uptake and lead to molecular changes, including altered gene expression, proteome and metabolome, not only in the root itself but also in the shoot. In addition, fine-tuning of directional root growth is affected, reducing the efficiency of root maneuvers. Finally, recent studies have shown that direct illumination masks phenotypes or alter the responses of the experiments performed. The application of tools that protect roots from direct light illumination will lead to results that are closer to natural plant responses and will allow a more reliable transfer of knowledge from basic to applied plant science. Since most of the research data obtained so far come from studies with roots exposed to light and fed exogenous sugars, we will make surprising revelations and discover new connections between signaling pathways in the future, but overall, these findings will help us to better understand how roots and plants adapt to ever-changing environmental conditions.

## Data Availability

No new data were created or analyzed in this study. Data sharing is not applicable to this article.
